# Maltohexaose-indocyanine green (MH-ICG) for near infrared imaging of endocarditis

**DOI:** 10.1371/journal.pone.0247673

**Published:** 2021-03-01

**Authors:** Kiyoko Takemiya, Joachim J. Røise, Maomao He, Chung Taing, Alexander G. Rodriguez, Niren Murthy, Mark M. Goodman, W. Robert Taylor

**Affiliations:** 1 Division of Cardiology, Department of Medicine, Emory University School of Medicine, Atlanta, Georgia, United states of America; 2 Department of Bioengineering, University of California at Berkeley, Berkeley, California, United States of America; 3 Department of Chemistry, University of California at Berkeley, Berkeley, California, United States of America; 4 Department of Radiology and Imaging Sciences, Emory Center for Systems Imaging, Emory University School of Medicine, Atlanta, Georgia, United states of America; 5 Cardiology Division, Atlanta Veterans Affairs Medical Center, Atlanta, Georgia, United states of America; 6 Department of Biomedical Engineering, Emory University School of Medicine and Georgia Institute of Technology, Atlanta, Georgia, United states of America; Babasaheb Bhimrao Ambedkar University (A Central University), INDIA

## Abstract

Infectious endocarditis is a life-threatening disease, and diagnostics are urgently needed to accurately diagnose this disease especially in the case of prosthetic valve endocarditis. We show here that maltohexaose conjugated to indocyanine green (MH-ICG) can detect *Staphylococcus aureus* (*S*. *aureus*) infection in a rat model of infective endocarditis. The affinity of MH-ICG to *S*. *aureus* was determined and had a Km and Vmax of 5.4 μM and 3.0 X 10^−6^ μmol/minutes/10^8^ CFU, respectively. MH-ICG had no detectable toxicity to mammalian cells at concentrations as high as 100 μM. The *in vivo* efficiency of MH-ICG in rats was evaluated using a right heart endocarditis model, and the accumulation of MH-ICG in the bacterial vegetations was 2.5 ± 0.2 times higher than that in the control left ventricular wall. The biological half-life of MH-ICG in healthy rats was 14.0 ± 1.3 minutes, and approximately 50% of injected MH-ICG was excreted into the feces after 24 hours. These data demonstrate that MH-ICG was internalized by bacteria with high specificity and that MH-ICG specifically accumulated in bacterial vegetations in a rat model of endocarditis. These results demonstrate the potential efficacy of this agent in the detection of infective endocarditis.

## Introduction

Infectious endocarditis (IE) is a systemic infection in which bacteria form vegetations on the heart valves. The diagnosis of IE is made based on the modified Duke criteria in which the involvement of endocardium or supporting tissue is one of the major diagnostic criteria [1]. Transthoracic echocardiography is widely accepted as an approach to assist in the diagnosis of IE. However, there are limitations to this imaging modality. Disadvantages include limited visualization due to body habitus and imaging in the setting of a prosthetic valve in which there are artifacts that limit visualization of the valve structures [2]. While transesophageal echocardiography improves the diagnostic accuracy in many cases, it has limitations related to imaging; it is also a semi-invasive procedure. Echocardiography is also of limited utility in its ability to determine whether the bacterial infection in the heart has been eliminated by antibiotic therapy. Therefore, there is an unmet need to develop more sensitive and less invasive imaging modalities to assist in the diagnosis and management of IE.

To develop novel diagnostic methods that detect the existence of bacteria in the tissue and organs, we have focused on the differences in sugar uptake by bacteria and mammalian cells [3–5]. Bacteria have maltodextrin transporters that actively internalize maltodextrins as an energy source; mammalian cells do not. Of particular importance to our approach is that the maltodextrin transporter can accept maltodextrins that bear structural modifications such as the addition of fluorescent tags [3, 6–8]. Therefore, maltodextrin-based imaging probes conjugated with fluorescent dyes and radionuclides, such as the positron emitter fluorine-18, can detect the existence of bacteria in the body with high sensitivity and specificity [3–5]. We recently showed that maltohexaose-based imaging probes—[F-18]fluoro-maltohexaose for positron emission tomography (PET) imaging and maltohexaose-IR786 for near infrared fluorescent imaging—are potentially useful to detect pacemaker pocket infections in a rat model [4]. We also observed that the accumulation of maltohexaose-based imaging probes is very low in the heart; other studies also indicated low accumulation of maltodextrin analogues in the heart [3, 4, 9–13]. These findings suggested the potential utility of maltohexaose-based imaging probes for the detection of IE. Maltohexaose-IR786 has a strong fluorescent signal that is detectable with clinical near infrared fluorescent imaging devices, but there is still a concern of potential cytotoxicity. Therefore, we developed a second generation maltohexaose-based imaging probe, maltohexaose-indocyanine green (MH-ICG). ICG is a near infrared fluorescent dye with low toxicity that is in clinical use and is excreted via the liver without being modified [14]. Near infrared wavelength light is permeable to soft tissue and blood for up to a few centimeters, and ICG is clinically used for vascular imaging and lymph node detection [15]. As a derivative of ICG, MH-ICG is detectable with current near infrared imaging devices for clinical use, and is therefore a promising candidate for determining the extent of bacterial infection. In this study, we evaluated the basic characteristics of MH-ICG *in vitro* and *in vivo* and whether MH-ICG is accumulated in bacterial vegetations in a rat model of endocarditis.

## Materials and methods

All animal protocols were approved by the Emory University Institutional Animal Care and Use Committee along with the guidelines and rules of Association for Assessment and Accreditation of Laboratory Animal Care International (AAALAC International). All animal experiments were conducted in accordance with an approved protocol (PROTO201700806).

### Synthesis of maltohexaose-indocyanine green (MH-ICG)

**Fig 1:**
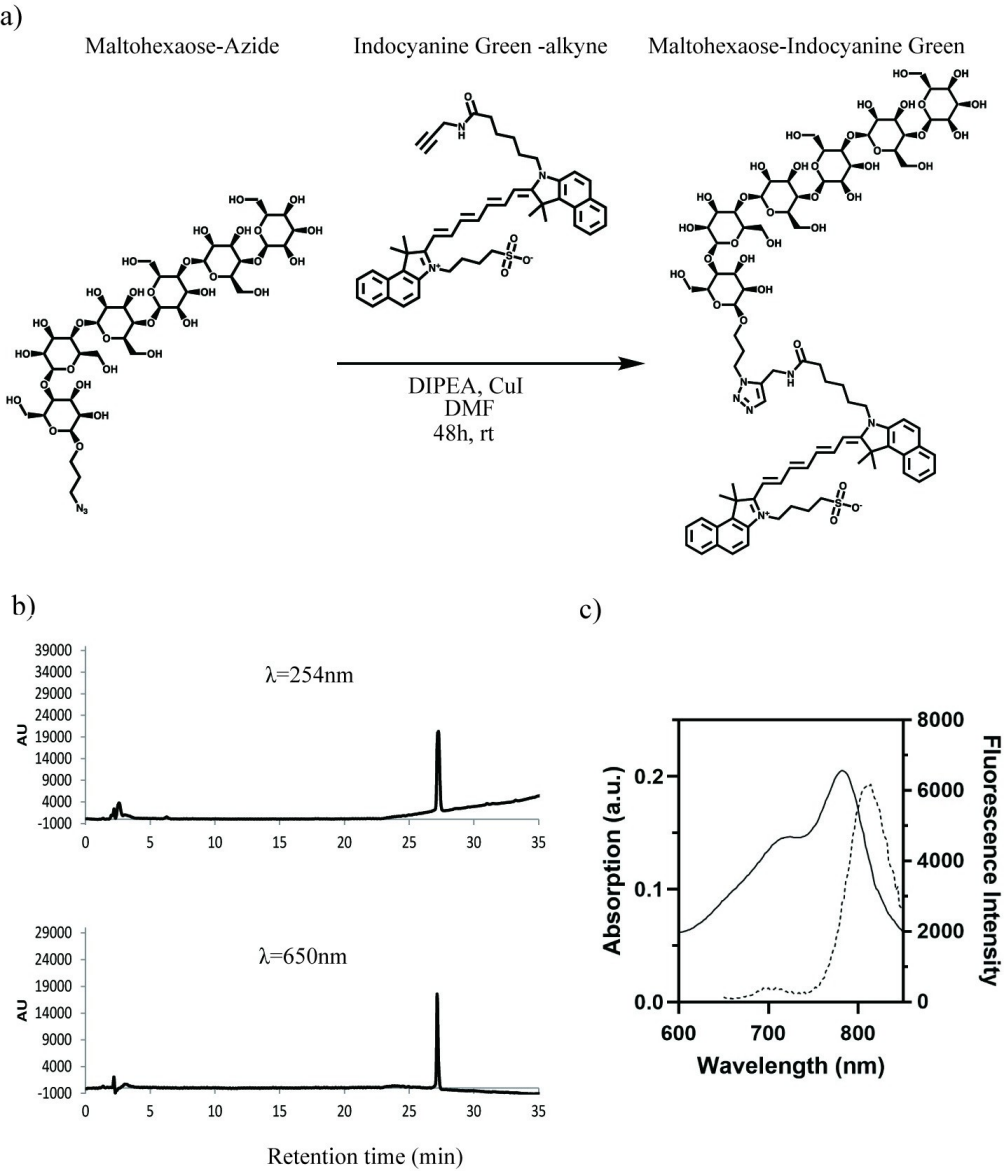
Synthesis of MH-ICG. a) Maltohexaose-azide was conjugated with ICG-alkyne through a copper-catalyzed click reaction. b) Analytical HPLC confirmed the purity of MH-ICG. Purified MH-ICG was injected onto an analytical Xbridge C18 5 μm 4.6 x 150 mm column. (Flow rate: 1 ml/min. Solvent system: MeCN/H_2_O + 0.1% TFA, 0–20 min: 40% MeCN, 20–40 min: gradient to 100% MeCN.) Product eluted at 27.2 min. c) Fluorescence spectrum of MH-ICG. The absorbance and fluorescence intensities are indicated as solid and broken lines, respectively. The emission spectrum was generated with an excitation wavelength of 760 nm.

MH-ICG was synthesized through a copper-catalyzed click reaction with MH-azide [3] (25 mg, 0.023 mmol, 1 eq) and commercially available ICG-alkyne (21.5 mg, 0.028 mmol, 1.2 eq, obtained from Iris biotech GMBH) in the presence of DIPEA (1 mg, 7.6 μmol, 0.33 eq) and CuI in catalytic amounts (Fig 1A). After mixing in DMF (0.01 M to ICG-alkyne) at room temperature for 48 hours, the product was diluted in DI H_2_O (1:9) and filtered before purification using HPLC on a XBridge Prep C8 5 um OBD, 19 x 150 mm semi-prep column (flow rate: 10 ml/min. Solvent system: MeCN/H_2_O + 0.1% TFA, 0–20 min: 37% MeCN, 20–50 min: gradient to 100% MeCN; the product eluted at 27 min.). The resulting MH-ICG was analyzed using mass spectrometry (ESI^+^: found: 1863.7391m/z, calc: 1863.7408m/z for [C_87_H_120_O_35_N_6_SNa^+^], S1 Fig), and the purity was assessed by analytical HPLC (Fig 1B). The spectrofluorimetric properties of MH-ICG were assessed, and the absorption and emission maxima were observed at 782 nm and 814 nm, respectively, with emission being measured following excitation at 760 nm. (Fig 1C). We used 760 and 830 nm as the excitation and emission wavelengths, respectively.

### Cytotoxicity assay

CHO-K1 cells (ATCC CCL-61) cultured in F-12K medium with 10% FBS were used to evaluate the cytotoxicity of MH-ICG. On day -1, the cells were seeded in 96 well plates at density of 1 X 10^3^ cells/well. On day 0, the cells were loaded with 0, 2.5, 10, 25, 50, or 100 μM of MH-ICG, and the cells were cultured for 24 and 72 hours. On days 1 and 3, the medium with MH-ICG was changed to 100 μl/well of fresh F-12K medium followed by 20 μl/well of a tetrazolium compound-based reagent to evaluate the cell viability (3-(4,5-dimethylthiazol-2-yl)-5-(3-carboxymethoxyphenyl)-2-(4-sulfophenyl)-2H-tetrazolium, inner salt; MTS, CellTiter 96 AQueous One Solution Cell Proliferation Assay, Promega). The cells were incubated at 37°C for 60 minutes, and the formazan generated was measured at 490 nm with a microplate reader (Varioskan LUX, Thermo Fisher Scientific). The viability of MH-ICG treated cells was calculated as [absorbance of MH-ICG loaded cells]/[absorbance of control cells] (n = 4 /each concentration).

### Uptake of MH-ICG by bacteria

In this study, we used *Staphylococcus aureus* (*S aureus*, ATCC 25923; Gram-positive bacteria) because *S*. *aureus* is one of the most commonly observed pathogens in patients with IE [1, 2, 16, 17]. To evaluate the affinity of MH-ICG for *S*. *aureus*, the bacteria at a density of 1 X 10^8^ colony forming units (CFU)/ml were cultured with MH-ICG at concentrations of 0, 2.5, 5, 10, 20, and 30 μM for 1 hour at 37°C. The bacteria in 1 ml of culture were then washed in phosphate buffered saline (PBS) 3 times and re-suspended in 100 μl of PBS. The amount of MH-ICG internalized by bacteria was evaluated with a fluorescent microplate reader (Varioskan LUX, Thermo Fisher Scientific) (n = 3/each concentration).

### Right heart IE model

Male Sprague Dawley rats (SD rats) weighing 250 to 275g were obtained from Charles River Laboratories. The animals were anesthetized with 1–2% isoflurane, and plastic catheters were inserted into the right ventricle via the right jugular vein. The location of the catheter was determined by the backflow of blood. The proximal end of the catheter was connected to a vascular access port (Braintree Scientific, Inc, U.S.A.) implanted on the back, and the incision was closed. On post-operative day 1 (POD 1), the rats were injected with 1 X 10^8^ CFU/0.1 ml of *S*. *aureus* via the tail vein. The rats were used for experiments on POD 3 (two days after inoculation of *S*. *aureus*) when a vegetation had formed around the catheter in the right ventricle. Control rats did not receive catheterization nor inoculation of bacteria. (n = 5/each group). The rats were euthanized with carbon dioxide after the experiments.

### Quantification of bacteria in the right ventricle

On POD 3, the right heart infectious endocarditis model rats (IE rats) were sacrificed with carbon dioxide, and the heart was extracted aseptically. The heart was cut in pieces and placed in Luria-Bertani (LB) broth. The bacteria in the heart were extracted and suspended in LB broth using a TissueLyser II (Qiagen). Serial dilutions of the suspensions were cultured on LB agar plates to determine the number of bacteria in the hearts.

### Accumulation of MH-ICG in vivo

On POD 3, the IE rats were injected with 0.25 ml of 1 mM MH-ICG via the tail vein. Rats without catheterization and inoculation were used as controls. The rats were sacrificed 4 hours after injection of MH-ICG, and the hearts were sliced in three segments and scanned with an *in vivo* imaging device (In-Vivo Xtreme, Bruker). The fluorescent intensity in the vegetation and that in the left ventricle free wall were measured in each slice, and the mean intensity ratio defined as [the mean intensity of the vegetation]/ [the mean intensity of the left ventricle free wall] was calculated for each slice. The average of the intensity ratio in 3 slices was used as the intensity ratio of the sample. In the control samples, the mean intensity in the right ventricle free wall was used instead of that of the vegetation.

### Biological half-life of MH-ICG

Male SD rats weighing 250 to 275 g were used for this experiment. The animals were anesthetized with 1–2% isoflurane and injected with 0.25 ml of 1 mM MH-ICG via the tail vein. At 15 and 30 minutes as well as 1, 2, 4, and 6 hours after injection of MH-ICG, 100 μl of blood was sampled with a heparinized syringe from the lateral tail vein opposite to the one used for injection of MH-ICG (n = 4 /each timepoint). The blood samples were centrifuged at 5000 X g for 10 minutes, and the plasma concentrations of MH-ICG were evaluated by a near infrared fluorescent microplate reader (Varioskan LUX, Thermo Fisher Scientific). The biological half-life of MH-ICG was calculated based on a two-compartment model with the following formula.

Cn+1=Cn*(1/2)^[(tn+1‐tn)/T]

Where Cn indicates the plasma concentration of MH-ICG in the sample n, tn indicates the sampled time point, and T indicates the biological half-life. Based on this formula, the biologil half-life in each animal and each time point was calculated.

### Biodistribution of MH-ICG

Male SD rats weighing 180 g to 190 g were injected with 0.25 ml of 1 mM MH-ICG via the tail vein and were housed in metabolic cages to collect feces and urine. The rats were sacrificed 24 hours after injection of MH-ICG, and the organs and tissue were harvested. The samples were homogenized with RIPA buffer using a TissueLyser II (Qiagen). The concentrations of MH-ICG in samples were evaluated with a fluorescent microplate reader (Varioskan LUX, Thermo Fisher Scientific) (n = 4).

### Statistical analysis

Analysis was performed with Prism statistical software (GraphPad Software). Student’s t-test was used for comparison of two groups, and P<0.05 was considered to be significant. All data are shown as mean ± standard error.

## Results

### Basic characteristics of MH-ICG

**Fig 2:**
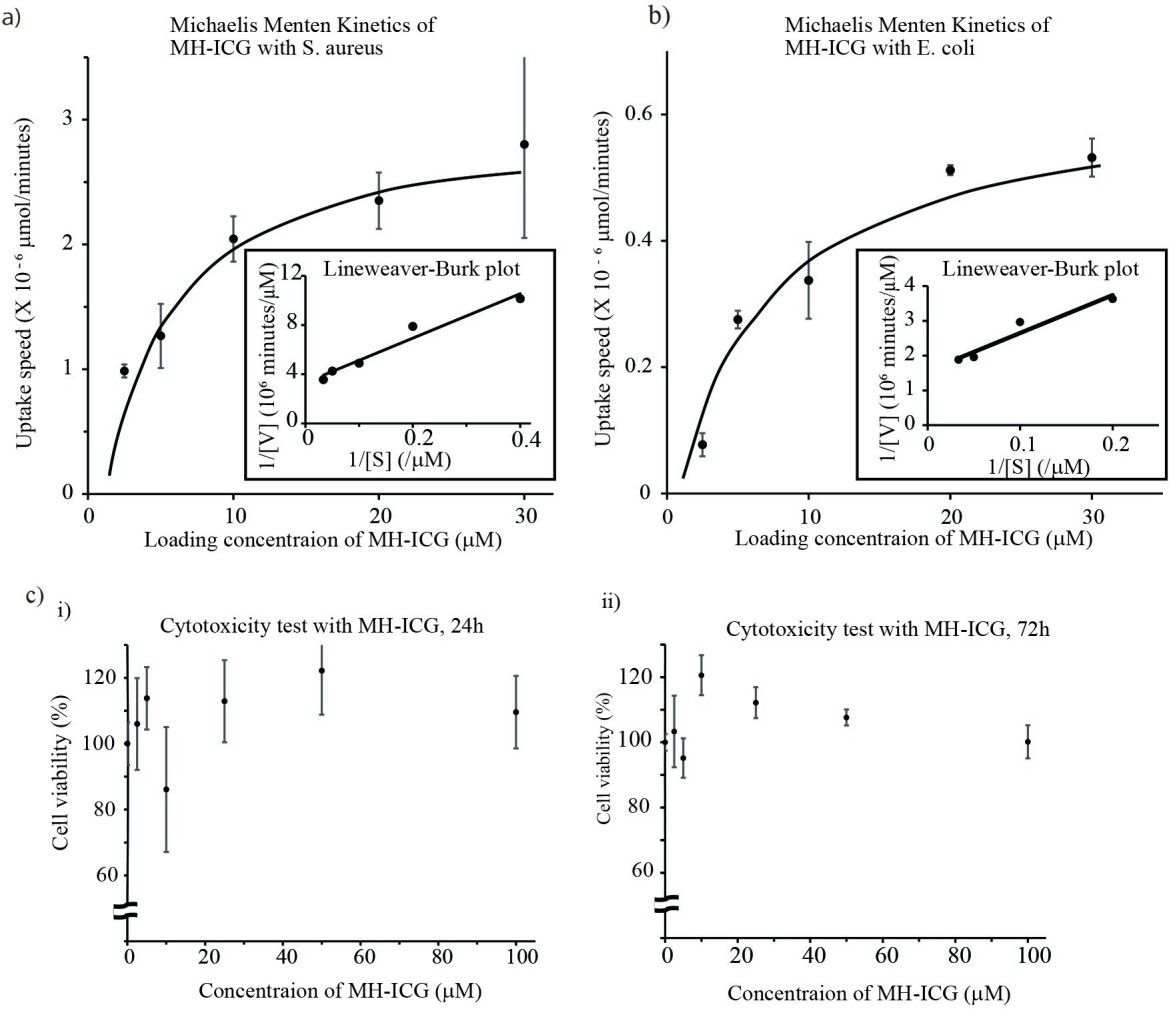
Basic characteristics of MH-ICG in vitro. To evaluate the uptake of MH-ICG in bacteria, *S*. *aureus*, *E*. *coli*, and LamB mutant *E*. *coli* were cultured with MH-ICG at various concentrations (n = 3 /each concentration). The internalized amount of MH-ICG by *S*. *aureus* (a) and *E*. *coli* (b) followed Michaelis-Menten kinetics. The Km and Vmax calculated with a Lineweaver-Burk plot were 5.4 μM and 3.0 X 10^−6^ μmol/minutes/10^8^ CFU for *S aureus* and 6.9 μM and 6.3 X 10^−7^ μmol/minutes/10^8^ CFU for *E*. *coli*, respectively. LamB mutant *E*. *coli* internalized a small amount of MH-ICG at the level of detection. c) Cytotoxicity of MH-ICG was evaluated in CHO-K1 cells. The cell viability was evaluated for 24 hours (i) and 72 hours (ii) after loading of MH-ICG. The cells were incubated with 0 to 100 μM of MH-ICG (n = 4 /each concentration), and no reduction in cell viability was observed up to 100 μM of MH-ICG at either time points.

We first evaluated the kinetics of MH-ICG internalization by *S*. *aureus*. The relationship between the concentrations of MH-ICG and the internalizing velocity of *S*. *aureus* is shown in Fig 2A. *S*. *aureus* internalized MH-ICG even at low concentrations, and the uptake velocity plateaued at a concentration of 20 μM. For 10^8^ CFU of *S*. *aureus*, the Km and Vmax calculated using a Lineweaver-Burk plot analysis were 5.4 μM and 3.0 X 10^−6^ μmol/minutes/10^8^CFU, respectively.

We also evaluated the kinetics of MH-ICG in *Escherichia*. *coli* (*E*. *coli*, ATCC 33456) and LamB mutant *E*. *coli* (JW-3996-1), which lacks maltoporin on the outer membrane [18]. As shown in Fig 2B, the Km and Vmax were 6.9 μM and 6.3 X 10^−7^ μmol/minutes for 10^8^ CFU of *E*. *coli*, respectively. The amount of MH-ICG internalized by 1 X 10^8^ CFU of LamB mutant *E*. *coli* was 25.1 ± 4.4 pmol using 30 μM MH-ICG compared to 19.7 ± 1.1 pmol internalized with 2.5 μM MH-ICG. The uptake of MH-ICG by LamB mutant *E*. *coli* did not follow Michaelis-Menten kinetics consistent with the loss of specific maltoporin-mediated uptake. These results demonstrate that MH-ICG uptake by bacteria is highly specific and occurs via the maltodextrin transport system.

The cytotoxicity of MH-ICG was evaluated in CHO-K1 cells. The MH-ICG showed no effects on cell growth or viability at concentrations of up to 100 μM for up to 3 days emphasizing the safety of MH-ICG for mammalian cells (Fig 2C).

### MH-ICG accumulates in bacterial vegetations

**Fig 3:**
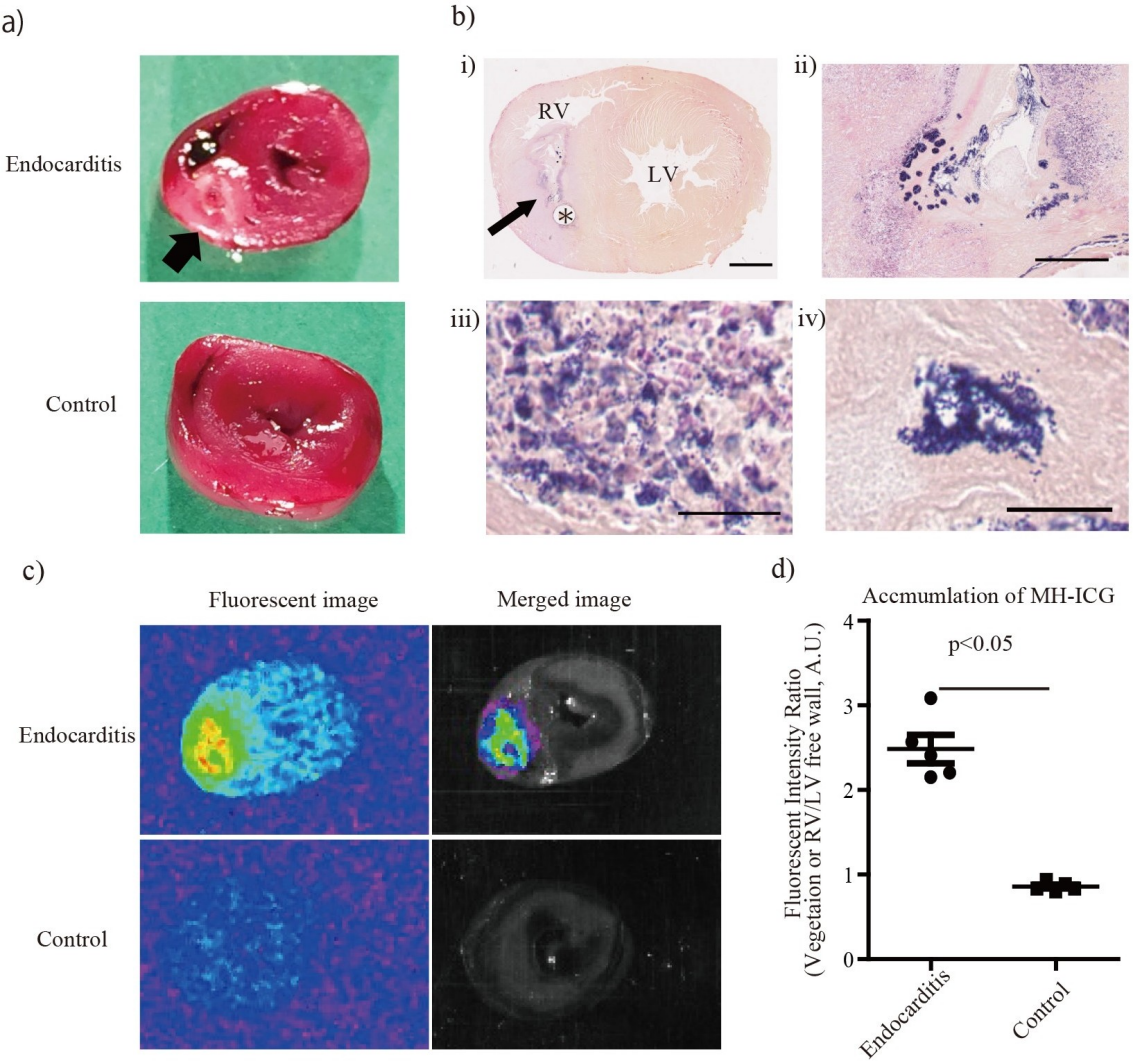
Accumulation of MH-ICG in vegetation. a) Right heart endocarditis was established by catheterizing in the right ventricle followed by injection of *S*. *aureus*. The vegetation was found around the catheter in the right ventricle (the arrow). b) i) Whole image of the specimen. Arrow: vegetation, *: the catheter in right ventricle, RV: right ventricle, LV: left ventricle. Bar: 2 mm. ii) Low power field of vegetation around the catheter. In the vegetation, spreading and accumulation of Gram-positive bacteria are observed in the fibrous tissue. Bar: 250 μm. iii) High-power field of vegetation. Gram-positive cocci are observed among large inflammatory cell infiltrates. Bar: 25 μm. iv) High-power field of vegetation with accumulation of bacteria. The deep purple spot is composed of Gram-positive cocci. Bar: 25 μm. c) Near infrared imaging with MH-ICG in the right heart endocarditis model in rats. Accumulation of MH-ICG was only observed in the vegetation. The hearts from the control rats had a very low fluorescent signal. d) Accumulation of MH-ICG in vegetation was quantified as an intensity ratio defined as [fluorescent intensity in vegetation or the right ventricle]/[fluorescent intensity in the left ventricle free wall]. The intensity ratio in IE rats was 2.5 ± 0.2, which was significantly increased versus control rats (n = 5 /group).

The rats developed right heart endocarditis two days after inoculation of *S*. *aureus* (Fig 3A). Histological evaluation with Gram staining confirmed the accumulation of Gram-positive cocci in vegetations (Fig 3B). Total bacterial counts in the myocardium were (1.3 ± 0.5) X 10^8^ CFU (n = 3).

To evaluate the accumulation of MH-ICG in the vegetations, the IE and control rats were injected with MH-ICG and sacrificed 4 hours after injection of MH-ICG. Fig 3C shows that MH-ICG accumulation was only observed only in the vegetation. The fluorescent intensity of MH-ICG accumulating in the vegetation was 2.5 ± 0.2 times higher than that in the left ventricle free wall, and this intensity ratio in the IE group was significantly increased versus the control group (Fig 3D).

### Plasma half-life and biodistribution of MH-ICG

**Fig 4:**
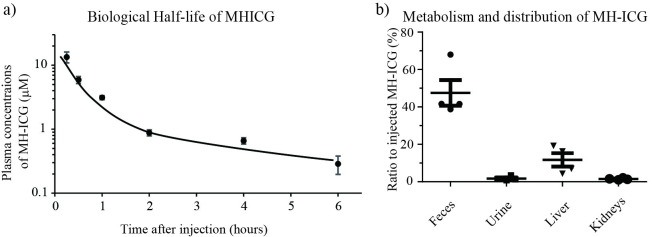
Distribution of MH-ICG *in vivo*. Healthy rats were injected with MH-ICG. a) The plasma concentration of MH-ICG was monitored for six hours. The plasma concentration was reduced very rapidly in two hours, and the biological half-life of MH-ICG in the initial phase was calculated as 14.0 ± 1.3 minutes (n = 4). Note that the concentration of MH-ICG is on a logarithmic scale, and some standard error bars overlap the average circles. b) Metabolism and distribution of MH-ICG were also evaluated: After 24 hours, about 50% of injected MH-ICG was excreted into feces. The MH-ICG that remained in the liver and in the kidneys were 11.7 ± 3.5% and 1.5 ± 0.2%, respectively (n = 4).

The pharmacokinetics of MH-ICG were evaluated in rats without infection. The MH-ICG rapidly cleared from the circulation with a pattern consistent with a dual mode of elimination (Fig 4A). The half-life of MH-ICG calculated from the plasma concentrations at 15 and 30 minutes was 14.0 ± 1.3 minutes; the half-life for the second phase from 15 and 60 minutes was 25.8 ± 3.8 minutes. Two hours after injection of MH-ICG, the plasma concentration was less than 5% compared with the plasma concentration of MH-ICG at 15 minutes. The plasma concentrations of MH-ICG decreased slowly after that. These results demonstrate that MH-ICG is cleared from the circulation very rapidly, which is desirable to minimize background fluorescence.

The biodistribution studies revealed that approximately 50% of the injected MH-ICG was excreted within 24 hours with the majority of the product in the feces (Fig 4B). The remaining MH-ICG was found in the liver (11.7 ± 3.5%) and kidneys (1.5 ± 0.2%). In the other organs, such as the heart, lungs, and intestine, less than 1% of the injected MH-ICG remained. The serum concentration of MH-ICG at 24 hours after injection was below the limit of detection. These results suggest that MH-ICG is primarily metabolized by the liver and excreted into the feces.

## Discussion

We report a novel and fluorescence-based imaging probe that can detect bacterial endocarditis lesions in a highly specific fashion. We used an *in vitro* assay system to demonstrate that MH-ICG is not taken up by mammalian cells and that uptake by bacteria largely occurs specifically through the maltodextrin transport system. The *in vivo* data confirm that systemically administered MH-ICG is effectively taken up by bacteria in our endocarditis model and that the free MH-ICG is effectively cleared from the circulation relatively quickly. The latter is important for both minimizing potential toxicity as well as decreasing background signal. In summary, these results support the concept that MH-ICG has the appropriate *in vivo* characteristics for localization of bacterial endocarditis.

The model of endocarditis that we employed of these studies is somewhat unique in that it is a model of right heart endocarditis. Several previous studies of experimental endocarditis have used an aortic valve endocarditis model [19–23]. We elected not to use aortic valve endocarditis because of the higher incidence of systemic embolism and the very high rate of mortality (up to 40% within 48 hours) [20]. The right heart model of endocarditis that we employed exhibited low mortality with all of the animals surviving until the studies were completed. In the right heart endocarditis model, the vegetation is formed around the catheter in the right ventricle and involves the tricuspid valve thus limiting hemodynamic compromise. The number of bacteria in our IE model was in the range of 10^8^ CFU, which is similar to that in the aortic valve endocarditis model [20–22].

MH-ICG is our second generation of maltohexaose-based fluorescent probe; it has ICG as the probe molecule instead of IR-786 used in our first generation of maltohexaose-based fluorescent probes [3, 4]. Both ICG and IR-786 are fluorescent cyanine dyes, but IR-786 consists of indolium and has a “rigidification ring” in the center of a polymethylene bridge. ICG consists of benz[e]indolium with sulfonate groups and has no “rigidification ring”. These differences affect the excitation and emission wavelengths as well as the stability and cytotoxicity of IR-786 and ICG [24]. Conceição et al. demonstrated that the cytotoxicity of polymethylene dyes on Hela cells is caused by the degradation products of these dyes, and the existence of the sulfonate groups and the “rigidification ring” do not affect the stability of these dyes [25]. They also report that the cytotoxicity of polymethylene dyes on Hela cells is related to the permeability of these dyes through the cell membrane [25]. Here, we showed that MH-ICG has lower cytotoxicity on CHO-K1 cells likely due to decreased permeability as a result of conjugation with maltohexaose. The half-life of ICG in aqueous solutions is about 17 hours [26]. This is sufficient for clinical use including bacterial imaging, but this shorter half-life of ICG might also contribute to a reduction in MH-ICG cytotoxicity.

The biodistribution studies were interesting in that approximately half of the injected MH-ICG was excreted into feces in 24 hours. This could reflect degradation of MH-ICG because ICG itself is known to be excreted into feces. Alternatively, the addition of ICG to MH might alter the clearance mechanism, i.e., ^18^F-MH is cleared primarily by the kidneys [4, 27].

The limitation of MH-ICG in diagnosis of endocarditis is the permeability of the near infrared wavelength. Similar to ICG, the penetration of excitation and emission wavelength of MH-ICG is limited to a few centimeters of tissue, and endocarditis lesions could not be imaged through the chest wall with MH-ICG. However, visualization of the extent of bacterial infiltration could be assessed during valve replacement and other surgeries for infectious lesions such as orthopedic device infection; similar fluorescence imaging is done in certain settings [15, 28–31]. Moreover, MH-ICG might be useful for bedside diagnosis of device infections near the skin such as pacemakers and left ventricular assist devices (LVADs) infections. In future, it is possible that the development of small near infrared wavelength detectors might enable the early diagnosis of endocarditis with a trans-esophageal approach. Finally, with proper gating, it is possible that PET-based imaging could provide the necessary resolution for detecting bacterial endocarditis using MH-based probe.

## Conclusion

In this study, we demonstrated that MH-ICG can determine the presence and extent of bacterial infection in the setting of endocarditis in a highly specific manner. MH-ICG has low cytotoxicity and is a viable candidate for further study of potential clinical applications; it may provide a unique diagnostic tool for bacterial endocarditis.

## Supporting information

S1 Fig (TIF)

S1 Data (DOCX)
