# Supplementary figures and images for: Maltohexaose-indocyanine green (MH-ICG) for near infrared imaging of endocarditis
Source: PLoS One. 2021 Mar 1;16(3):e0247673. doi: 10.1371/journal.pone.0247673 (PMC7920357; doi:10.1371/journal.pone.0247673)

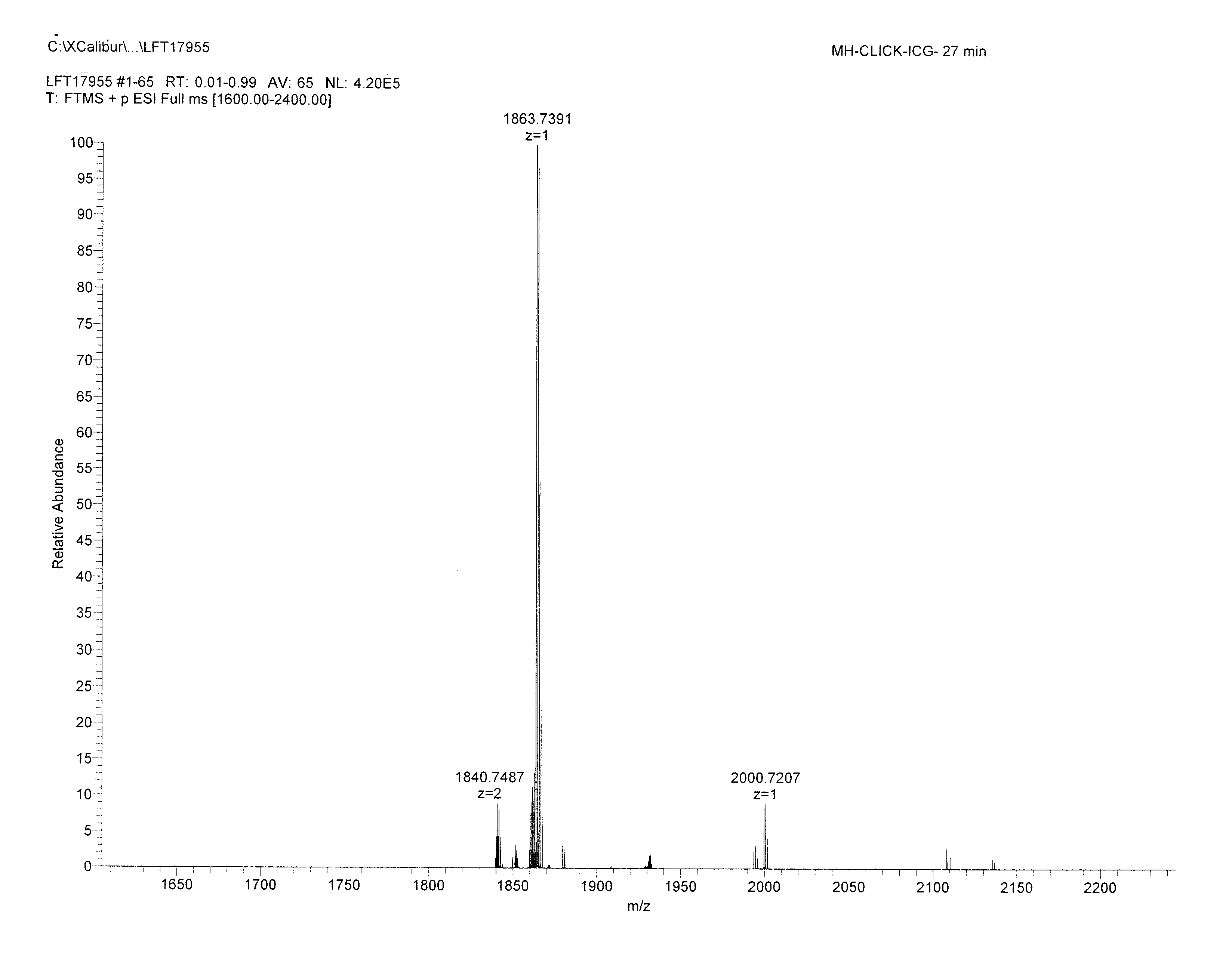

Supplement: S1 Fig — (TIF) [file pone.0247673.s001.tif]
