# Supplementary material for: Maltohexaose-indocyanine green (MH-ICG) for near infrared imaging of endocarditis
Source: PLoS One. 2021 Mar 1;16(3):e0247673. doi: 10.1371/journal.pone.0247673 (PMC7920357; doi:10.1371/journal.pone.0247673)
Supplement: S1 Data — (DOCX) [file pone.0247673.s002.docx]

**S1 Data.**

The data used to calculate averages and standard error, and not indicated in the manuscript or figures are indicated in this supporting data.

**Cytotoxicity of MH-ICG**

Cell viability at day 1 (24h) and at day 3 (72h)

| Day 1 viability (%) | Loading concentrations of MH-ICG (uM) | | | | | | |
| --- | --- | --- | --- | --- | --- | --- | --- |
|  | 0 | 2.5 | 5 | 10 | 25 | 50 | 100 |
| test 1 | 94.4 | 74.9 | 98.8 | 65.2 | 80.2 | 83.1 | 95.2 |
| test 2 | 92.2 | 92.8 | 96.4 | 66.4 | 109.9 | 136.5 | 88.4 |
| test 3 | 94 | 138.2 | 133.6 | 69.8 | 139.4 | 127.1 | 118.4 |
| test 4 | 119.5 | 118.1 | 126.3 | 143 | 122.2 | 142 | 136.5 |

| Day 3 viability (%) | Loading concentrations of MH-ICG (uM) | | | | | | |
| --- | --- | --- | --- | --- | --- | --- | --- |
|  | 0 | 2.5 | 5 | 10 | 25 | 50 | 100 |
| test 1 | 101.6 | 90.4 | 90.3 | 96.2 | 91.6 | 93.1 | 90.9 |
| test 2 | 94.5 | 96.7 | 109 | 120.6 | 125 | 108.6 | 97.8 |
| test 3 | 102.9 | 96.7 | 90.9 | 140.7 | 122.5 | 111.7 | 98.5 |
| test 4 | 101.1 | 129.6 | 90.6 | 124.8 | 109.7 | 117.1 | 113.4 |

**Uptake of MH-ICG by bacteria**

| Bacteria | Loading concentration of MH-ICG (uM) | | | | |
| --- | --- | --- | --- | --- | --- |
|  | 30 | 20 | 10 | 5 | 2.5 |
| S. aureus | 0.784 | 1.679 | 1.059 | 0.455 | 0.531 |
|  | 2.071 | 1.304 | 1.431 | 0.880 | 0.610 |
|  | 2.189 | 1.247 | 1.189 | 0.947 | 0.634 |
| E. coli | 0.333 | 0.299 | 0.197 | 0.17 | 0.032 |
|  | 0.261 | 0.315 | 0.100 | 0.165 | 0.046 |
|  | 0.363 | 0.307 | 0.311 | 0.160 | 0.061 |
| LamB mutant E. coli | 0.181 | 0.215 | 0.092 | 0.157 | 0.195 |
|  | 0.332 | 0.310 | 0.223 | 0.305 | 0.179 |
|  | 0.241 | 0.122 | 0.331 | 0.224 | 0.217 |

(uM of MH-ICG, 100ul of bacterial suspension)

**Quantification of bacteria in vegetation in the right ventricle of IE rats**

| Rat | CFU/heart |
| --- | --- |
| IE-1 | 2.0 X 10^8^ |
| IE-2 | 1.6 X 10^8^ |
| IE-3 | 0.3 X 10^8^ |

**Biological half-life of MH-ICG in healthy rats**

| Plasma concentrations of MH-ICG (uM) | Time after injection (hour) | | | | | |
| --- | --- | --- | --- | --- | --- | --- |
|  | 0.25 | 0.5 | 1 | 2 | 4 | 6 |
| H-rat1 | 3.594 | 0.145 | 0.814 | 0.600 | 0.501 | 0.033 |
| H-rat2 | 0.744 | 0.654 | 0.480 | 0.951 | 0.676 | 0.387 |
| H-rat3 | 0.860 | 0.954 | 0.635 | 0.029 | 0.859 | 0.448 |
| H-rat4 | 0.694 | 0.948 | 0.550 | 0.943 | 0.608 | 0.282 |
